# Comparative Study on the Engineering Performance of Lime- and Cement-Improved Argillaceous Siltstone

**DOI:** 10.3390/ma19112422

**Published:** 2026-06-05

**Authors:** Yi Chen, Fangcheng Huang, Rongcheng Zhan, Mengqi Zhou, Hui Weng, Hao Yang

**Affiliations:** 1 Key Laboratory for Highway Engineering of Ministry of Education, Changsha University of Science & Technology, Changsha 410114, China; 2 Zhejiang Communications Investment Construction Management Co., Ltd., Hangzhou 310000, China; 3 Zhejiang Communications Investment Group Expressway Construction and Management Co., Ltd., Hangzhou 310024, China; 4 School of Transportation, Changsha University of Science & Technology, Changsha 410114, China

**Keywords:** argillaceous siltstone, subgrade engineering, lime improvement, cement improvement, wet-dry cycles

## Abstract

**Highlights:**

- Cement-stabilized argillaceous siltstone achieves 1.5–1.7 times the strength of lime-stabilized soil at equal dosage, and retains 78.3% UCS after five wet–dry cycles versus 69.0% for lime soil, demonstrating superior durability
- Low-dosage (3%) lime-improved soil degrades to 47.5 kPa after cycling, falling below the minimum engineering usability threshold for subgrade applications.
- High-grade roadbeds should prioritize 7% cement improvement, while 7% lime is viable for general subgrade sections; low-dosage (<5%) lime is not recommended for argillaceous siltstone subgrades.

**Abstract:**

Argillaceous siltstone is widely distributed along expressways in southern China; however, its strong water sensitivity and slaking properties severely restrict its utilization as subgrade fill, particularly under wet–dry cyclic conditions where bearing capacity deteriorates sharply. Existing studies have predominantly focused on mechanical performance evaluation of stabilizers, while systematic comparisons of lime and cement improvement effects and durability evolution under wet–dry cycles remain insufficiently understood. Drawing on the Yongjin Expressway reconstruction and expansion project, this study systematically investigates the durability of lime- and cement-improved argillaceous siltstone fill. Through unconfined compressive strength (UCS) tests, California bearing ratio (CBR) tests, and five wetting–drying cycles, the evolution differences in strength development, water stability, and durability between the two improvement schemes are revealed. Results indicate that, under identical stabilizer contents (3–7%) and curing conditions, the UCS and CBR of cement-improved soil are significantly higher than those of lime-improved soil. At the same dosage, the strength of cement-improved soil is approximately 1.5–1.7 times that of lime-improved soil, and the absolute strength gap further widens with increasing dosage. Both stabilizers effectively inhibit water immersion swelling, but the swelling rate of lime-improved soil is about 1.3–1.5 times that of cement-improved soil at the same dosage. At 7% dosage, the swelling rates of cement- and lime-improved soils decrease to 0.40% and 0.60%, respectively, both meeting subgrade fill swelling control requirements. After five wet–dry cycles, the UCS retention rate of 7% cement-improved soil is 78.3%, while that of lime-improved soil is 69.0%; the residual strengths are 507.0 kPa and 303.6 kPa, respectively, both satisfying general subgrade engineering strength requirements. However, the 3% lime-improved soil declines to 47.5 kPa after cycling, falling below the engineering threshold. Integrating strength, deformation, and durability indices, high-grade highway roadbeds and other high-load-bearing sections should prioritize 7% cement improvement, whereas general subgrade sections and locations emphasizing crack resistance may adopt 7% lime improvement as an alternative. Low-dosage (<5%) lime improvement is not recommended for argillaceous siltstone subgrade engineering. The findings provide a scientific basis for the engineering application of argillaceous siltstone as subgrade fill and for optimization of improvement schemes.

## 1. Introduction

Argillaceous siltstone, as a weak rock-soil material widely distributed in southern China, poses severe technical challenges in expressway subgrade engineering due to its distinct bedding development and significant hydrophilicity. This type of rock is hand-breakable, rapidly softens and slakes upon water immersion, and accumulates internal structural damage under wet–dry cyclic action, leading to sharp deterioration of bearing capacity. Concurrently, its pronounced deformation characteristics—including high compressibility, notable swelling upon water immersion, and shrinkage cracking upon drying—further compromise subgrade stability and accelerate structural failure under cyclic environmental loads. This severely restricts its resource utilization in high-grade road projects. Taking the Yongjin Expressway reconstruction and expansion project as an example, the argillaceous siltstone widely distributed along the route, if directly used as subgrade fill, is highly prone to strength attenuation and excessive deformation during the service life, threatening long-term operational safety. Nevertheless, despite its poor engineering properties, the resource utilization of argillaceous siltstone as subgrade fill possesses full engineering necessity. If entirely excavated and discarded, not only will enormous transportation costs be incurred, but also substantial land resources will be occupied. Moreover, the Ministry of Transport’s Green Highway Construction Guide explicitly requires “adapting measures to local conditions and utilizing local materials,” promoting the large-scale application of solid wastes in transportation infrastructure [1,2]. As a major component of engineering waste residue, the resource utilization of argillaceous siltstone is an inevitable choice to achieve the “dual-carbon” goal of highway construction [3]. Therefore, suppressing its water sensitivity and enhancing engineering performance through chemical improvement has become the key technical path for the safe application of argillaceous siltstone in subgrade engineering. Deepening the understanding of its wet-dry cycle deterioration mechanisms, systematically evaluating chemical improvement effects, and establishing reliable mechanical performance evaluation systems have become critical scientific issues urgently to be solved in geotechnical engineering.

Around the slaking and deterioration mechanisms of argillaceous siltstone, scholars at home and abroad have carried out extensive fundamental research. Qi et al. [4] revealed the progressive failure mechanism of red-bed argillaceous siltstone during wet–dry cycles through slaking tests, identifying capillary stress fatigue and clay mineral swelling as the primary causes of rock mass structural failure. Gautam and Shakoor [5] systematically compared the slaking characteristics of clay-bearing rocks under laboratory and natural climatic conditions, pointing out that the traditional Slake Durability Index evaluation method cannot fully reflect the coupling effects of complex field environments. Liu and Zhang [6] established a quantitative slaking evaluation model for strongly weathered argillaceous siltstone under wet–dry cycles, confirming that surface fracture networks gradually connect with increasing cycle numbers, ultimately leading to significant macro-mechanical performance attenuation. On this basis, Yang et al. [7] further demonstrated through studies on carbonaceous argillaceous siltstone that internal micro-cracks significantly expand after wet–dry cycles, with scanning electron microscopy observations confirming the dissolution of cementing materials caused by hydraulic erosion. Wang et al. [8] revealed through wet–dry cycle tests on red-bed mudstone in the Three Gorges Reservoir area that the durability of the rock–mortar interface is the key factor controlling long-term subgrade stability. Tamrakar et al. [9] also showed in their study on the slaking characteristics of mudstone in the Nepalese Siwalik Group that the Slake Durability Index of mudstone is closely related to its mineral composition and cementation degree. In recent years, research has further extended to multi-factor coupled deterioration. Yu et al. [10] investigated the mechanical properties and microstructural alterations of silty mudstone under dry–wet cycling in acidic environments, revealing that porosity intensifies and coalesces from isolated pores to interconnected voids, with fractal dimensions quantitatively capturing the mesoscopic damage evolution. Similarly, Di [11] elucidated the mechanical degradation and acoustic emission characteristics of damaged mudstone, highlighting that cyclic stress disturbances lead to accelerated crack propagation and a shift from distributed small-scale cracking to concentrated large-scale fracture. Furthermore, Qi et al. [12] examined red-bed mudstone-based controlled low-strength materials and demonstrated that cement incorporation markedly enhances water stability under wet–dry cycles, with residual strength initially increasing during early cycles due to continued hydration, subsequently declining, and finally stabilizing after nine cycles. The aforementioned studies have revealed the deterioration laws of argillaceous siltstone under wet–dry cycles from different perspectives; however, they mainly focus on the natural slaking process of unimproved rock mass, with insufficient attention to the durability evolution of chemically improved rock-soil materials, making it difficult to provide direct theoretical support for improvement design.

In terms of chemical improvement technology, lime and cement, as two traditional stabilizers, exhibit essential differences in their reinforcement mechanisms and engineering effects. Pandey and Rabbani [13] reviewed the improvement mechanisms of lime- and cement-stabilized subgrade soils, pointing out that lime mainly functions through ion exchange and pozzolanic reactions, while cement relies on hydration products to form a spatial network structure. Dhar and Hussain [14] demonstrated that the strength development of lime-stabilized subgrade soil in road construction has a significant time effect, with long-term curing substantially enhancing its unconfined compressive strength. Keybondori and Abdi [15] also confirmed the effectiveness of lime improvement in reducing the plasticity index and improving workability in their study on clay-textured forest soil road subgrades. In contrast, Feng et al. [16] conducted a technical–economic comparative study of cement and lime improvement of sandy lean clay in Chinese expressway projects, showing that cement improvement offers better cost-effectiveness in enhancing subgrade bearing capacity. Okonkwo and Kennedy [17] evaluated the stabilization effects of cement and lime on high-plasticity expansive subgrade soils, finding that cement outperforms lime in controlling volume change and long-term stability. Saleh et al. [18] compared the improvement effects of cement, lime, and fly ash on subgrade soils, confirming that cement has significant advantages in short-term strength development. Recently, the incorporation of industrial solid wastes as supplementary stabilizers has attracted considerable attention. Sanijiya et al. [19] conducted a comprehensive study on copper slag blended with lime and cement for expansive subgrade stabilization, finding that cement-based combinations consistently outperformed lime-based mixes in wet–dry durability and strength, with SEM confirming denser calcium-silicate-hydrate (C-S-H) gel matrices. Ubani et al. [20] investigated cement-stabilized tropical clay–quarry dust mixtures, reporting that soaked CBR values reached up to 172% and UCS development was significantly enhanced by the filler and pozzolanic effects of quarry dust. Ozioko et al. [21] employed machine learning algorithms to predict CBR and compressibility of lime-stabilized lateritic soil, achieving R^2^ values exceeding 0.9997 and demonstrating that predictive modeling can effectively guide dosage optimization. Balcha et al. [22] explored barley husk ash as a supplementary pozzolanic material with lime for expansive subgrade soils, confirming that the combined treatment increased UCS by 111% and met subgrade strength requirements. Additionally, research on coal gangue–carbide slag (CS-CG) composite stabilizers [23] has revealed that industrial waste binders can achieve comparable mechanical properties and durability to traditional Portland cement, with XRD and SEM-EDS analyses elucidating the formation of C-S-H and C-A-H gels. These studies provide important references for the performance evaluation of lime- and cement-improved soils; however, they mostly concentrate on ordinary cohesive soils or sandy soils. Systematic comparative studies on lime and cement improvement of argillaceous siltstone—a specific lithology—during multiple wet–dry cycles remain insufficient, and the quantitative understanding of their improvement efficiency differences is still lacking.

In terms of mechanical performance evaluation, unconfined compressive strength (UCS) and California bearing ratio (CBR) are core indices for evaluating the engineering performance of improved soils. Kardani et al. [24] established a prediction model for the UCS of unsaturated cemented soils using evolutionary algorithms, revealing the coupled influence mechanism of water content and cement dosage on strength. Soltani et al. [25] proposed a theoretical description framework for the UCS of artificially cemented fine-grained soils through dimensional analysis, emphasizing the nonlinear relationship between curing time and strength development. Baldovino et al. [26] studied the long-term strength evolution of lime-improved silty soil, showing that after 180 days of curing, UCS could reach more than 2.5 times the 28-day strength, reflecting the delayed strengthening effect of pozzolanic reactions. Teodoru et al. [27] and Al Bodour et al. [28] respectively employed machine learning and gene expression programming to establish UCS prediction models for cement-treated soils based on fundamental soil parameters, providing new technical means for strength estimation. In CBR evaluation, Katte et al. [29] statistically analyzed the correlation between CBR and the physical–mechanical properties of subgrade soils, establishing a CBR prediction equation based on liquid limit and plasticity index. Naeini and Gholampoor [30] studied the effects of wet–dry cycles and plasticity index on the CBR of lime-stabilized clay, finding that CBR retention rates after five wet-dry cycles were generally below 40%. Abbey et al. [31] showed in their study on sustainable stabilization materials for expansive subgrade soils that increasing wet–dry cycle numbers led to significant reductions in CBR and resilient modulus for all specimens. Ahmed et al. [32] and Lillian et al. [33] respectively expanded the research perspectives on CBR evaluation of roadbed materials from the angles of recycled material utilization and multi-index comprehensive evaluation. Recent advances have further integrated microstructural characterization with macro-mechanical performance. Ubani et al. [20] correlated the resilient modulus and CBR of cement-stabilized tropical clay–quarry dust mixtures with pore structure evolution, while Ozioko et al. [21] demonstrated that machine learning-based CBR prediction models can achieve exceptional accuracy when trained with expanded datasets using Piecewise Cubic Hermite Interpolation. Although the above scholars have achieved fruitful results in the mechanical performance evaluation of improved soils, systematic comparative studies on the improvement effects of lime and cement on argillaceous siltstone under wet–dry cycle conditions remain insufficient. In particular, the differences in slaking inhibition mechanisms, strength evolution laws, and micro-mechanisms between the two stabilizers have not been systematically elucidated.

Despite the fruitful achievements outlined above, several critical gaps persist in the current understanding of chemically improved argillaceous siltstone under wet–dry cyclic conditions. While the natural slaking process of unimproved rock mass has been extensively documented, the durability evolution of chemically improved argillaceous siltstone—particularly the quantitative inhibition mechanisms of stabilizers on slaking characteristics under multiple wet–dry cycles—remains insufficiently elucidated. Recent studies on red-bed mudstone-based controlled low-strength materials and silty mudstone degradation have highlighted the severity of wet–dry damage, yet direct applications to subgrade fill stabilization with traditional lime and cement are still lacking. Furthermore, although lime and cement stabilization has been widely investigated for ordinary cohesive and sandy soils, systematic comparative evaluations targeting argillaceous siltstone as a specific lithology are scarce. Emerging research on industrial by-product stabilization and low-carbon binder systems has demonstrated promising alternatives, but a unified understanding of strength development, deformation characteristics, and water stability differences between traditional lime and cement for this specific rock-soil material is still absent. Most importantly, the differential damage mechanisms between lime-improved and cement-improved soils under wet–dry cycles, and their corresponding microstructural evolutions—such as cementation product types, spatial structure characteristics, and pore network deterioration—have not been deeply revealed at the quantitative level. Although SEM and XRD analyses have been employed in recent studies on stabilized soils, these micro-scale insights have rarely been correlated with macroscopic mechanical performance degradation in a systematic manner for argillaceous siltstone subgrades. Consequently, existing literature falls short of providing direct theoretical support and scientific bases for optimization of improvement schemes under different engineering scenarios, particularly for high-grade highway projects in southern China where argillaceous siltstone is abundantly distributed and wet–dry cyclic conditions are prevalent.

In view of this, drawing on the Yongjin Expressway reconstruction and expansion project, this study systematically investigates the durability comparison of lime- and cement-improved argillaceous siltstone fill. Through unconfined compressive strength (UCS) tests, California bearing ratio (CBR) tests, and five wetting–drying cycles, the differences in strength development, deformation characteristics, water stability performance, and durability evolution between the two improvement schemes are quantitatively revealed. Based on stress–strain curve morphology analysis, the brittle failure characteristics of cement-improved soil and the plastic failure characteristics of lime-improved soil and their engineering significance are elucidated. Through immersion swelling rate comparison, the volume stability of the two improved soils is evaluated. Combined with strength retention rate analysis after wet–dry cycles, optimization suggestions for improvement schemes under different engineering scenarios are proposed. The research findings provide a scientific basis for the safe application of argillaceous siltstone in high-grade road projects and possess important theoretical value and engineering significance for improving soft rock improvement design theory and guiding durability design of subgrade engineering.

## 2. Materials and Methods

### 2.1. Test Materials

**Figure 1:**
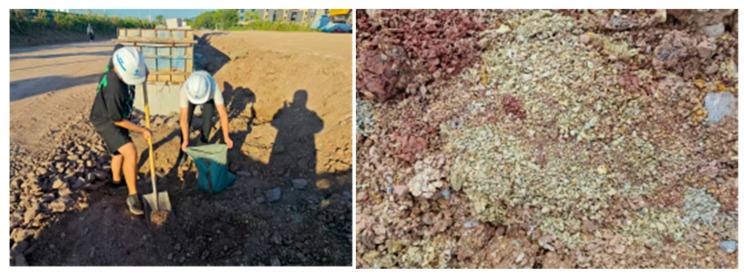
Sampling of argillaceous siltstone.

The test argillaceous siltstone was sampled from fill sections at K177+300, K178+700, and K181+100 of the Yongjin Expressway reconstruction and expansion project (Jinhua section, TJ03 bid section). Fresh rock samples are gray-brown, with well-developed bedding, hand-breakable, and rapidly soften and slake upon water immersion. Figure 1 illustrates the field sampling process of the argillaceous siltstone at the construction site. According to the *Test Methods of Soils for Highway Engineering* (JTG 3430-2020) [34], basic physical and mechanical property tests were conducted on intact rock samples, with results shown in Table 1.

According to the *Test Methods of Rock for Highway Engineering* (JTG 3431-2024) [35] and the *Technical Specification for Design and Construction of Highway Soft Rock Embankment* [36], slaking durability tests and slaking morphology tests were conducted on the obtained argillaceous siltstone, determining that it belongs to Class E soft rock (Class I easily slaking rock). For Class E rock, after pretreatment (sprinkling water for slaking followed by raking and compaction), it can be embanked using fill-soil techniques. When the CBR value after slaking cannot meet requirements, cement, lime, or other modifiers can be added for improvement. The cement used in this study was Conch brand P.O 42.5 ordinary Portland cement (Anhui Conch Cement Company Limited, Wuhu, China), a commonly used general-purpose silicate cement in Chinese highway engineering.

### 2.2. Test Scheme Design

To systematically study the effects of stabilizer type and dosage on the engineering performance of argillaceous siltstone, the test scheme shown in Table 2 was designed. Lime and cement dosages are expressed as percentages of dry soil mass, with four dosage levels set: 0% (control group), 3%, 5%, and 7% [37,38]. For each group, compaction tests, unconfined compressive strength tests, and CBR tests were conducted. Each CBR and UCS test group comprised six parallel specimens, cured for 7 days in an environment of 20 °C and 94% humidity.

#### 2.2.1. Wetting–Drying Cycles

**Figure 2:**
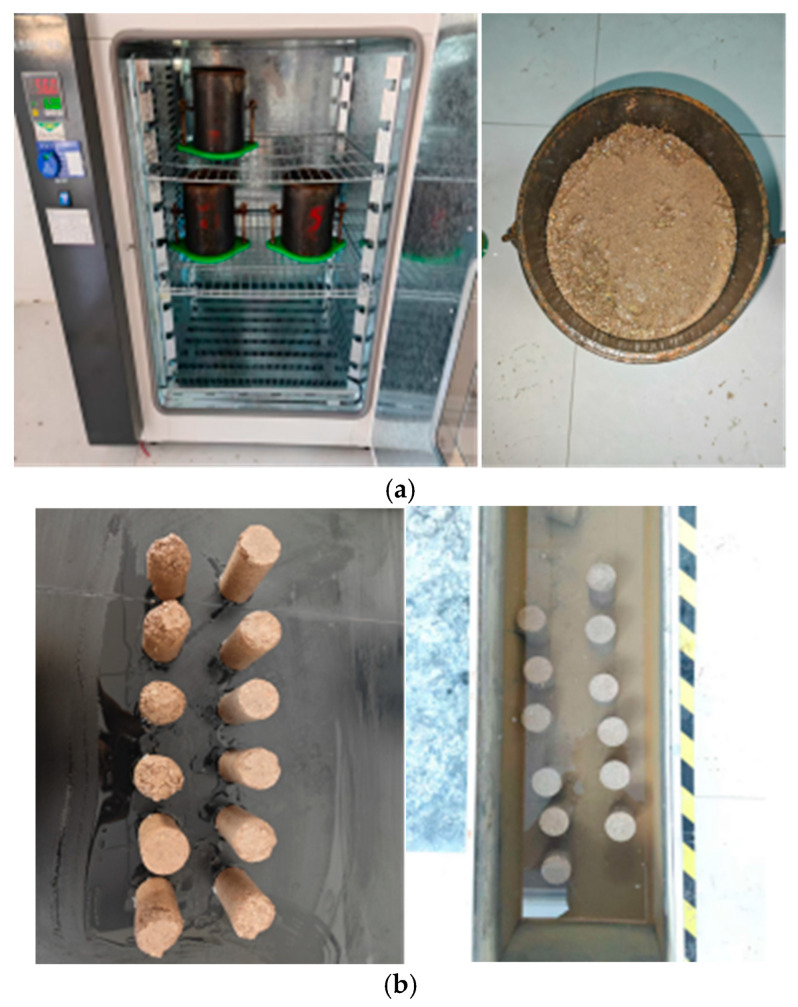
Wet–dry cycle experimental procedure: (**a**) CBR specimen wet–dry cycles; (**b**) UCS specimen wet–dry cycles.

Following the wet–dry cycle resilient modulus test method in the *Test Methods of Soils for Highway Engineering* (JTG 3430-2020) [34], argillaceous siltstone specimens designated for UCS and CBR tests were prepared. For each test condition (stabilizer type, dosage, and wet-dry cycle state), three parallel specimens were compacted to 96% of the maximum dry density, yielding average bulk densities (wet densities) ranging from 1.93 to 2.23 g/cm^3^ across the different mixture groups (Table 2). These specimens were then subjected to desorption and moisture absorption (soaking for 24 h, oven drying at 60 °C for 24 h (DHG-9070A, Shanghai Yiheng Scientific Instruments, Shanghai, China)), repeated for five cycles, as shown in Figure 2.

#### 2.2.2. Unconfined Compressive Strength Test

**Figure 3:**
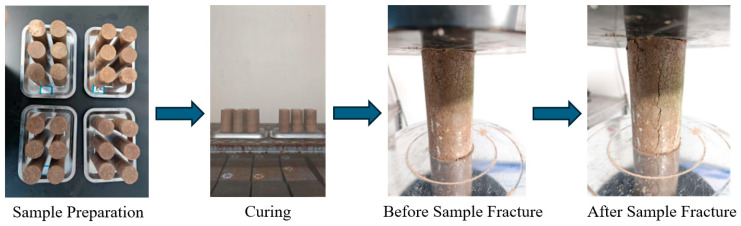
Unconfined compressive strength test.

To evaluate the unconfined compressive strength (UCS) of cement-improved argillaceous siltstone specimens, UCS tests were conducted according to the *Test Methods of Soils for Highway Engineering* (JTG 3430-2020) [34]. The specimen preparation process was as follows: First, argillaceous siltstone was dried and crushed, then passed through a 5 mm sieve. Second, water was added to the dried argillaceous siltstone at the optimum moisture content of each group, and the mixture was sealed for 24 h. Then, the moistened argillaceous siltstone was uniformly mixed with cement, compacted in three layers in a sample mold to prepare cylindrical specimens with a diameter of 50 mm, height of 100 mm, and compaction degree of 96% (Figure 3). The specimens were cured for 7 days at 20 °C and 94% humidity. After curing, tests were conducted using a universal testing machine (WDW-100, Jinan Shijin Group, Jinan, China) with a loading rate of 1 mm/min until specimen failure. During the test, stress–strain curves were collected and plotted in real time; the peak stress represents the unconfined compressive strength under the test conditions. Three parallel specimens were prepared for each test condition, and the average value was taken.

#### 2.2.3. California Bearing Ratio (CBR) Test

**Figure 4:**
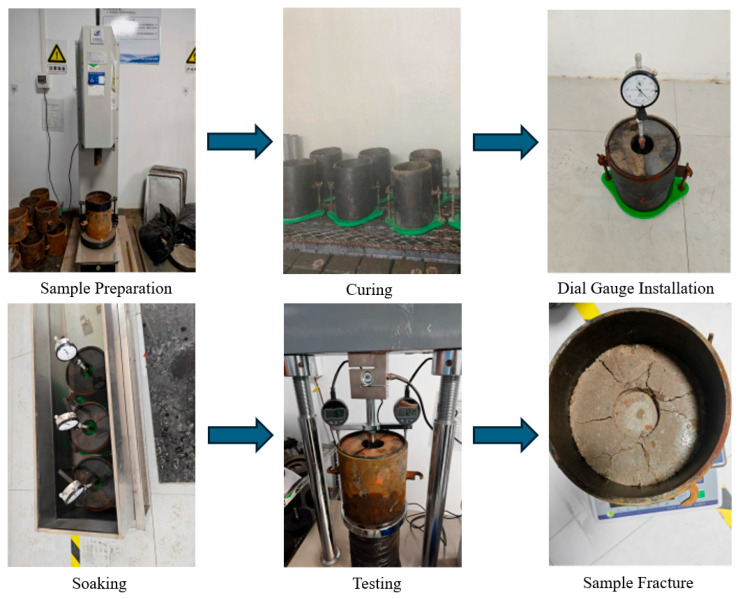
CBR test procedure.

CBR tests were conducted according to the standard [34]. The control particle size was 20 mm, and the specimen dimensions were 150 mm in diameter and 120 mm in height. The remaining specimen preparation process was consistent with that of the UCS test specimens. After curing, specimens were soaked in a constant-temperature water tank for 4 days, and penetration tests were conducted using a pavement strength tester (Figure 4) (Kailuode Instrument, Huanggang, China) equipped with a 50 kN load cell (accuracy ± 0.01 kN) and a linear variable differential transformer (LVDT) with a 50 mm stroke and 0.01 mm resolution. Real-time data from both sensors were logged via a dynamic data acquisition system at a sampling frequency of 10 Hz. The test was terminated when the specimen penetration reached 550 × 10^−2^ mm. Three parallel specimens were prepared for each test condition, and the average value was taken.

During the test, unit stress *p* versus penetration *l* curves were collected and plotted in real time. CBR_2.5_ is the ratio of unit pressure at 2.5 mm penetration to the standard load intensity of standard crushed stone at the same penetration, calculated according to Equation (1); CBR_5.0_ at *l* = 5 mm is simultaneously calculated according to Equation (2). If CBR_2.5_ < CBR_5.0_, the test is repeated; if the repeated result remains the same, the bearing ratio at *l* = 5 mm is adopted.

(1) $${CBR}_{2.5}=\frac{p}{7000}\times 100$$

(2) $${CBR}_{5.0}=\frac{p}{10,500}\times 100$$

where CBR is the California bearing ratio, calculated to 0.1%; *p* is the unit pressure (kPa). The larger of the two values is taken as the bearing ratio of the material.

According to the measured specimen height change in the CBR immersion test, the immersion swelling rate *γ* is calculated according to Equation (3):

(3) $$\gamma =\frac{\left(d_{1}-d_{0}\right)}{h}\times 100$$

where *d*_1_ is the final dial gauge reading after 4 days of immersion (mm); *d*_0_ is the initial dial gauge reading before immersion (mm); and *h* is the original specimen height, *h* = 120 mm.

## 3. Results and Discussion

### 3.1. Unconfined Compressive Strength

#### 3.1.1. Strength Development and Improvement Efficiency

**Figure 5:**
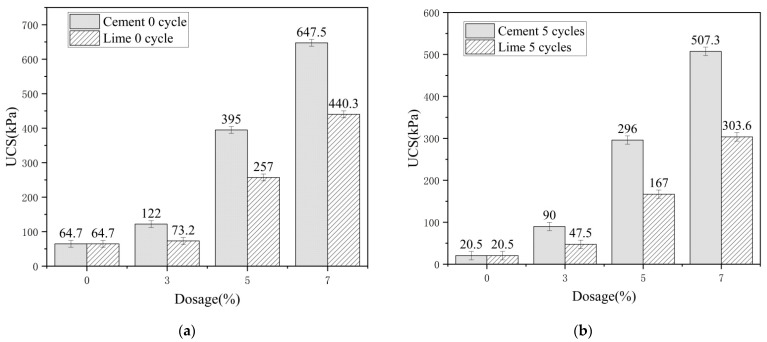
Unconfined compressive strength of improved argillaceous siltstone at different dosages: (**a**) 0 wet–dry cycles; (**b**) 5 wet–dry cycles.

After standard curing for 7 days, the UCS of cement- and lime-improved argillaceous siltstone exhibited significant differences and distinct dosage–response patterns compared with the untreated control (Figure 5). Under zero wet–dry cycles, the plain argillaceous siltstone (0% dosage) recorded a UCS of 55 kPa, serving as the baseline for evaluating improvement efficiency.

For cement-improved soil, the UCS increased approximately linearly with dosage: 122 kPa at 3% dosage, 395 kPa at 5% dosage, and 647.5 kPa at 7% dosage, representing an overall increase of 1077% relative to the untreated material. In contrast, lime-improved soil grew relatively gently: 73.2 kPa at 3% dosage, 257 kPa at 5% dosage, and 440.3 kPa at 7% dosage, an overall increase of 701%. Although lime improvement showed a higher relative increase from 3% to 7%, its absolute strength remained significantly lower than that of cement-improved soil at the same dosage, and its absolute gain over the control was markedly inferior to cement.

**Figure 6:**
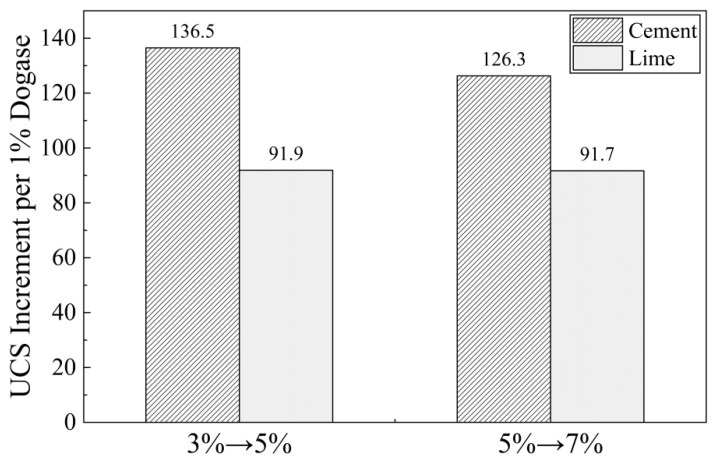
Incremental efficiency of unconfined compressive strength.

From the perspective of cumulative improvement efficiency (i.e., strength gain per unit dosage relative to the control, Figure 6), cement improvement demonstrated a progressively escalating efficiency: 22.3 kPa/% at 3% dosage, rising to 68.0 kPa/% at 5%, and reaching 84.6 kPa/% at 7%. This indicates that cement hydration not only provides immediate early strength but also sustains and accelerates its marginal contribution as dosage increases. Conversely, lime improvement exhibited a lower initial efficiency of 6.1 kPa/% at 3% dosage, which improved to 40.4 kPa/% at 5% and 55.0 kPa/% at 7%, suggesting that pozzolanic reactions require a threshold dosage to become fully effective and remain consistently below cement hydration in cost-effectiveness per unit additive.

Examining interval incremental efficiency (Figure 6), the UCS incremental efficiency of cement in the 3–5% range was 136.5 kPa/%, slightly decreasing to 126.3 kPa/% in the 5–7% range, indicating that cement hydration maintains a relatively high strength contribution efficiency at high dosages, though with slightly diminishing marginal effects. Lime improvement showed nearly constant incremental efficiencies of 91.9 kPa/% and 91.7 kPa/% in the two ranges, confirming that the strength enhancement rate of pozzolanic reactions remains relatively stable but is far lower than the strength increase rate of cement hydration.

Under identical dosage conditions, the UCS of cement-improved soil was approximately 1.47–1.67 times that of lime-improved soil, with the ratio decreasing as dosage increased: 1.67 at 3% dosage, decreasing to 1.47 at 7% dosage. This convergence suggests that while cement’s relative advantage is most pronounced at low dosages, increasing lime dosage can narrow the relative strength gap to a certain extent; however, it cannot close the absolute gap originating from the control baseline.

#### 3.1.2. Stress–Strain Characteristics

**Figure 7:**
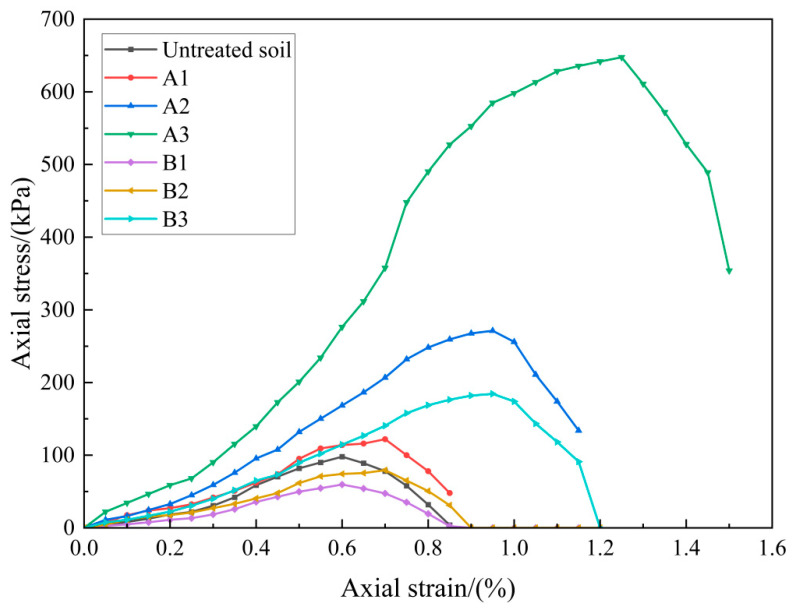
Stress–strain curve comparison at 0 wet–dry cycles.

Stress–strain curve morphology reveals the fundamentally different mechanical behaviors of the two improved soils (Figure 7). Cement-improved soil exhibits typical brittle failure characteristics: stress rapidly reaches the peak with increasing strain, with small peak strain (0.6–0.8%), and stress drops sharply after the peak, with low residual strength. Taking 7% dosage as an example, the peak stress of 647.5 kPa corresponds to a peak strain of 0.8%; after the peak, stress attenuates to below 30% of the peak strength with only an additional 0.4% strain.

The initial slope of the stress–strain curve for cement-improved soil is markedly steeper than that of lime-improved soil at equivalent dosages. Specifically, specimen A3 (7% lime) exhibits a significantly lower initial slope compared to its cement counterpart (B3), indicating reduced stiffness and more gradual stress accumulation. Although the slope of A3 increases moderately relative to A1 and A2 due to the higher lime content, it remains distinctly gentler than that of any cement-improved specimen, reflecting the inherent ductility of the lime-stabilized matrix.

In contrast, lime-improved soil shows obvious plastic failure characteristics: the stress–strain curve is gentler, with larger peak strain (0.8–1.1%), and stress attenuates progressively after the peak, with high residual strength. For 7% dosage lime-improved soil, after the peak strain of 1.1%, even when strain increases to 2.0%, the stress remains above 60% of the peak strength. This ductility characteristic means that lime-improved soil retains significant deformation energy dissipation capacity after reaching the ultimate bearing state, which is of important engineering significance for accommodating subgrade differential settlement and repeated traffic loading.

**Figure 8:**
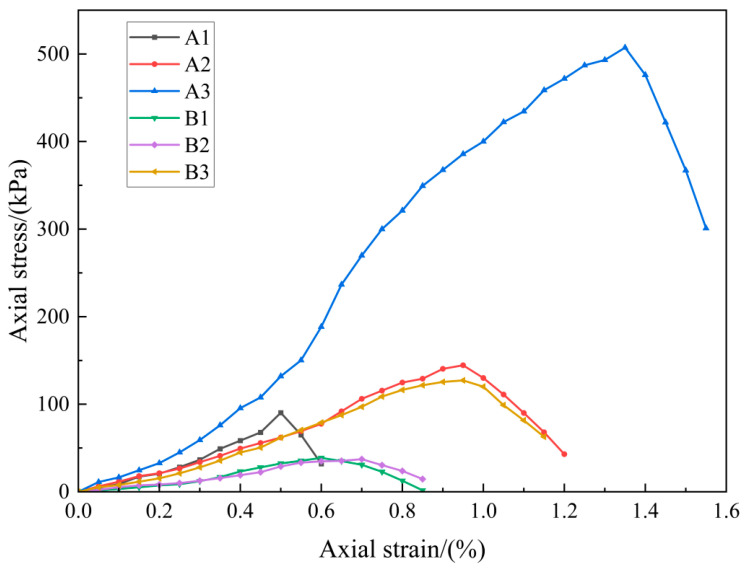
Stress–strain curve comparison after 5 wet–dry cycles.

After five wet–dry cycles, the stress–strain curves of both improved soils shift downward overall, but the morphological characteristics remain unchanged (Figure 8): cement-improved soil still maintains brittle failure mode, and lime-improved soil still maintains plastic failure mode. And the distinct difference in slope between A3 and the cement-improved specimens persists even after five wet–dry cycles, underscoring the fundamental contrast in cementation rigidity between the two stabilization systems. This indicates that wet–dry cycles mainly cause strength damage without altering the essential cementation structure types formed by the interaction between the two stabilizers and argillaceous siltstone.

**Figure 9:**
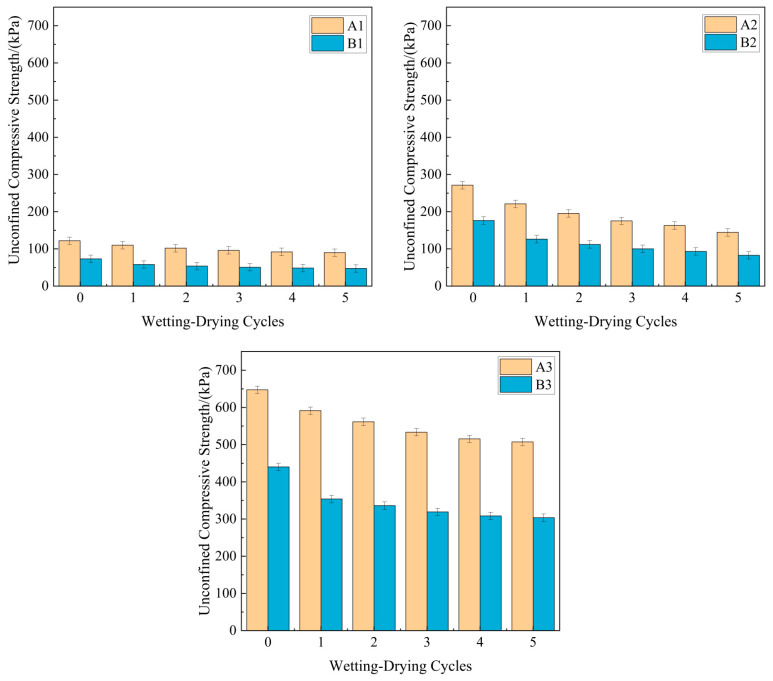
Comparison of UCS of cement- and lime-improved argillaceous siltstone.

Figure 9 presents the UCS evolution of cement- and lime-improved argillaceous siltstone under wet–dry cycles. Notably, under 3% dosage, the durability gap between the two improved soils is most significant: cement-improved soil still maintains 90.0 kPa UCS after five cycles, while lime-improved soil drops to 47.5 kPa, approaching the lower limit of the engineering usability threshold. Under 7% dosage, the gap between the lime-improved soil UCS retention rate of 69.0% and the cement-improved soil retention rate of 78.3% narrows to 9.3 percentage points, and the residual strength reaches 303.6 kPa. Nabil et al. [37] studied the wet–dry cycle durability of lime-stabilized soil, showing that low-dosage (<4%) lime-improved soil completely loses strength after cycling, while high-dosage (6–8%) lime-improved soil strength retention rates can increase to 25–40% with curing age. In this study, after five cycles, the 7% lime-improved soil residual strength was 303.6 kPa, falling within the described high-dosage lime-improved soil strength retention range, while the 3% dosage lime-improved soil after cycling was only 47.5 kPa, approaching the low-dosage failure threshold, indicating insufficient long-term stability and unsuitability for engineering practice.

### 3.2. California Bearing Ratio Characteristics

#### 3.2.1. CBR Improvement Effect and Dosage Response

**Figure 10:**
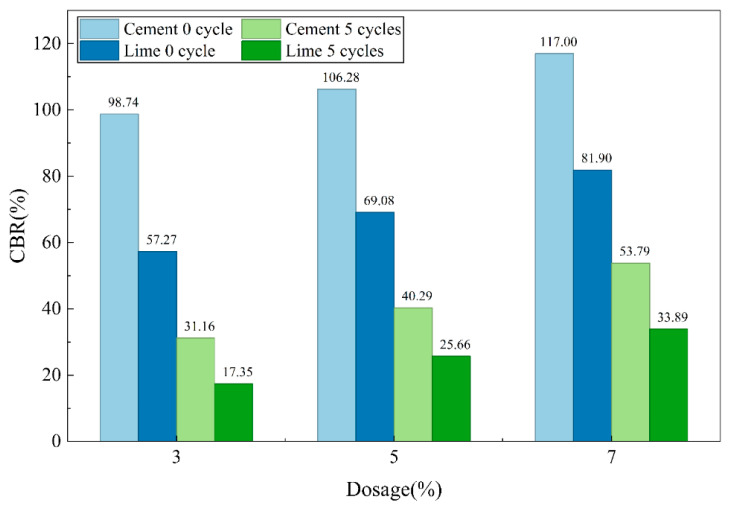
Comparison of CBR values after improvement with different stabilizers and dosages.

CBR test results show good consistency with UCS test results, further verifying the performance differences between the two stabilizers (Figure 10). Under zero cycles, 3% cement-improved soil CBR reached 98.74%, already meeting the technical requirements for high-grade highway roadbed fill (CBR ≥ 80%); lime-improved soil required 7% dosage to reach the equivalent level of 81.90%. The CBR of cement-improved soil increased steadily with dosage.

After five wet–dry cycles, the CBR of both improved soils underwent significant attenuation, and the attenuation amplitude was obviously greater than that of UCS under the same dosage conditions. For cement-improved soil, 3% dosage CBR decreased from 98.74% to 47.4%, with a retention rate of 48.0%; 7% dosage decreased from 138.5% to 63.7%, with a retention rate of 46.0%. The overall CBR retention rate of lime-improved soil was lower than that of cement-improved soil: 3% dosage decreased from 42.0% to 12.6%, with a retention rate of only 30.0%; 7% dosage decreased from 81.90% to 32.8%, with a retention rate of 40.0%.

From CBR incremental efficiency analysis, the CBR incremental efficiency of cement improvement in the 5–7% range is higher than that in the 3–5% range, showing an increasing marginal effect trend, while lime improvement CBR incremental efficiency remains relatively stable at 5.91%/% and 6.41%/%, and is slightly higher than cement in the high-dosage range. This indicates that the CBR contribution of lime improvement has better sustainability under high-dosage conditions, possibly related to the delayed strengthening effect of pozzolanic reactions.

#### 3.2.2. Evolution Characteristics of Penetration Resistance

**Figure 11:**
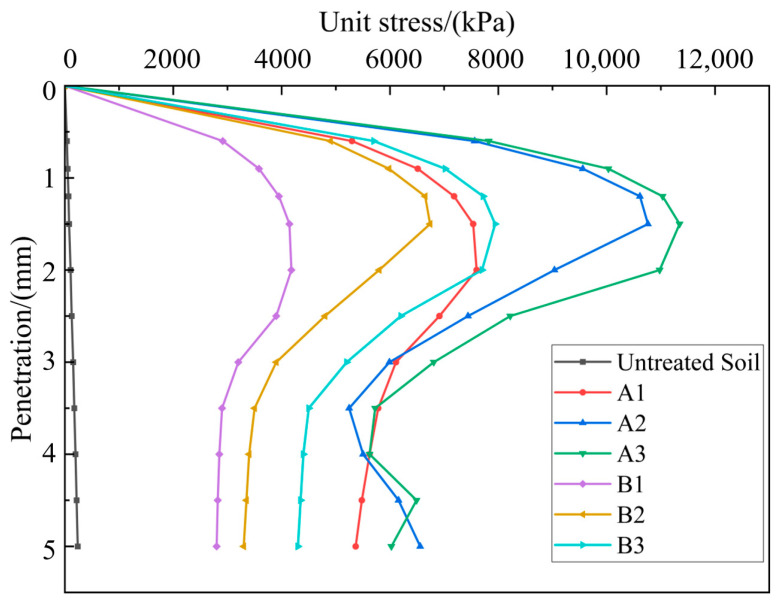
Comparison of CBR penetration curves.

The morphology differences of CBR penetration curves (*p*-*l* curves) reflect the structural response mechanisms of the two improved soils under loading (Figure 11). The *p*-*l* curve of cement-improved soil has a larger slope in the initial stage (penetration 0–1.5 mm), with rapid penetration resistance growth, indicating that it relies on rigid cementation structure to provide bearing capacity; however, after penetration exceeds 2.5 mm, the curve tends to flatten, and penetration resistance growth stagnates or even decreases, reflecting that once the rigid structure cracks, bearing capacity is rapidly lost.

The *p*-*l* curve of lime-improved soil has a relatively smaller slope, but penetration resistance shows a sustained and stable increasing trend with penetration depth, maintaining relatively high unit pressure in the 2.5–5.0 mm penetration range. This is related to the better plastic deformation capacity and interparticle friction-bite force of lime-improved soil, and also implies that its anti-fatigue performance under actual repeated traffic loading may be superior to that of cement-improved soil.

### 3.3. Swelling Characteristics

**Figure 12:**
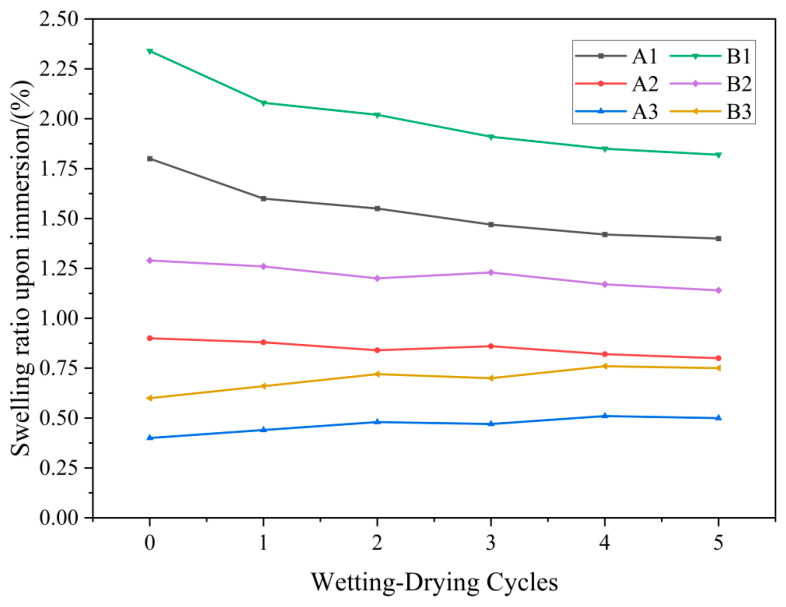
Comparison of immersion swelling rates under different stabilizers and dosages.

Argillaceous siltstone is rich in hydrophilic clay minerals, which easily produce swelling deformation upon water immersion. Previous studies have extensively documented the swelling characteristics of argillaceous siltstone and the inhibitory effects of chemical stabilizers. For example, Okonkwo and Kennedy [17] evaluated the effectiveness of cement and lime in controlling volume change and swelling potential of high-plasticity subgrade soils, while Abbey et al. [31] examined the wet–dry cycle behavior of expansive subgrade soils treated with sustainable cementitious materials. Additionally, Yu et al. [38] specifically investigated the swelling deformation of red-bed mudstone under cyclic wetting-drying conditions, reporting free swelling rates of 2.5–4.0% for untreated rock, which were substantially mitigated by cement-based stabilization. Both cement and lime improvement can effectively inhibit swelling, but the improvement effects differ significantly (Figure 12).

Under zero cycles, the swelling rate of lime-improved soil is higher than that of cement-improved soil at the same dosage: at 3% dosage, lime soil (2.34%) is 1.30 times that of cement soil (1.80%); at 5% dosage, it is 1.43 times; and at 7% dosage, it is 1.50 times. This indicates that the volume stability of lime-improved soil under immersion conditions is slightly inferior to that of cement-improved soil.

**Figure 13:**
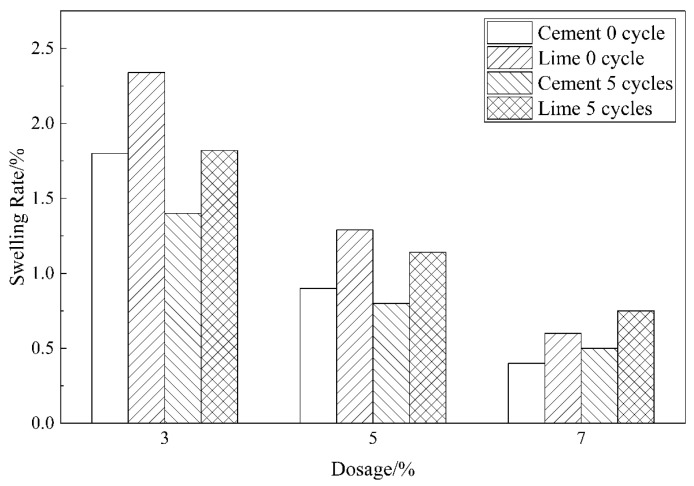
Comparison of two improved soils before and after wet–dry cycles.

The filling effect of cement hydration products on pores and the formation of dense cementation structures effectively block water intrusion and the hydration swelling of soil particles; while lime improvement initially relies mainly on ion exchange, the filling effect of cementation products is relatively weak, and lime itself has certain water absorption swelling characteristics, leading to slightly poorer swelling inhibition capacity. However, as dosage increases, the swelling rates of both improved soils show significant downward trends. At 7% dosage, the swelling rates of cement soil and lime soil decrease to 0.40% and 0.60%, respectively, both in the low swelling range, indicating that cement and lime improvement have significant inhibitory effects on the volume stability of argillaceous siltstone. Figure 13 presents a visual comparison of the two improved soils before and after the wet–dry cycles, further demonstrating the structural integrity and volume stability differences between cement- and lime-improved argillaceous siltstone under cyclic wetting–drying conditions.

### 3.4. Analysis of Improvement Mechanisms

Based on the essential differences in macro-mechanical behavior between the two improved soils—the brittle failure of cement-improved soil and the plastic ductile failure of lime-improved soil—combined with established conclusions from existing literature, reverse deduction and demonstration are conducted from three aspects: cementation product types, spatial structure characteristics, and wet–dry cycle damage mechanisms.

#### 3.4.1. Rigid Cementation Mechanism of Cement Improvement

After mixing cement with argillaceous siltstone, cement clinker minerals (C_3_S, C_2_S) rapidly undergo hydration reactions, generating calcium-silicate-hydrate (C-S-H) gel, calcium hydroxide (Ca(OH)_2_) crystals, and a small amount of ettringite (AFt). SEM observations by Horpibulsuk et al. [39] showed that cement hydration products first nucleate on soil particle surfaces, then grow into pores, forming dense spatial network structures that encapsulate and cement dispersed soil particles into larger composite clusters. This cementation structure dominated by C-S-H gel has high stiffness and bonding strength, but limited deformation capacity. Once external loads exceed its ultimate strength, micro-cracks rapidly initiate and connect in the brittle cementation layer, leading to sharp peaks and low residual strength in stress–strain curves. In this study, the peak strain of cement-improved soil was only 0.6–0.8%, and strength rapidly dropped below 30% after the peak, which is the macroscopic manifestation of sudden failure of the rigid cementation structure.

Furthermore, the significant pore-filling effect of cement hydration products reduces soil porosity, forming a relatively dense barrier layer that effectively blocks water intrusion and the hydration swelling of clay minerals. Consoli et al. [40] pointed out that cement hydration gel transforms loose particles into rigid monoliths through “physical filling–chemical cementation” dual action, which explains why the swelling rate of cement-improved soil in this study was consistently lower than that of lime-improved soil at the same dosage.

#### 3.4.2. Progressive Cementation Mechanism of Lime Improvement

The mechanism of lime improvement has obvious time effects and stage characteristics. Little [41] summarized lime stabilization reactions into four basic stages: ion exchange, flocculation–agglomeration, pozzolanic reaction, and carbonation. In the initial stage (hours to days), Ca^2+^ undergoes ion exchange with exchangeable cations (such as Na^+^, K^+^) on soil particle surfaces, causing clay particles to transform from flaky aggregation to flocculent structures, reducing plasticity and improving workability. Subsequently (days to months), under high pH conditions, active silico-aluminum minerals in soil undergo pozzolanic reactions with lime, generating C-S-H gel and C-A-H gel similar to cement hydration products, but the reaction rate is far slower than cement hydration, and product distribution is more dispersed [42].

This “ion exchange first, then pozzolanic reaction” progressive cementation mode means that lime-improved soil does not form a completely dense spatial skeleton within the 7-day curing period, and particles retain certain relative slip capacity. Therefore, lime-improved soil shows obvious plastic deformation characteristics under loading: gentle stress–strain curves, larger peak strain (0.8–1.1%), and residual strength after failure maintaining above 60% of peak strength. This ductility characteristic is of important engineering significance for accommodating subgrade differential settlement and repeated traffic loading. Baldovino et al. [26] also confirmed that lime-improved silty soil after 180 days of long-term curing can reach UCS more than 2.5 times the 28-day strength, reflecting the delayed strengthening effect of pozzolanic reactions.

#### 3.4.3. Differential Damage of Wet-Dry Cycles on Two Cementation Structures

The action of wet–dry cycles on improved soil is essentially a repeated process of “hydraulic erosion–cementation dissolution–structure reorganization.” For cement-improved soil, its rigid cementation structure under internal stresses generated by drying shrinkage and wet swelling, once micro-cracks initiate, rapidly expand along the brittle cementation layer, forming connected fracture networks, leading to rapid loss of cementation area. In this study, although the UCS retention rate of cement-improved soil after five cycles reached 78.3%, the CBR retention rate was only 46.0%, indicating that rigid structure damage is more severe under saturated conditions—the softening effect of water on cement paste pores and the leaching of Ca(OH)_2_ significantly weaken interparticle bonding [43].

For lime-improved soil, its relatively flexible cementation structure shows certain “self-adaptive” capability during wet–dry cycles: the flocculent structure formed by ion exchange can undergo particle rearrangement during wet swelling to release stress, and flocculent bodies shrink during drying without generating connected fractures. However, the total amount of pozzolanic reaction products in lime-improved soil is smaller and the cementation strength is lower; after multiple wet–dry cycles, the cumulative effect of cementation dissolution and particle detachment caused by hydraulic erosion is significant, manifested as overall lower strength retention rates than cement-improved soil. Notably, this study observed that the swelling rate of lime-improved soil decreased after five cycles (e.g., 3% dosage decreased from 2.34% to 1.82%), which may be related to wet–dry alternation promoting the full progress of ion exchange and the continuous development of a small amount of pozzolanic reactions, making the soil structure tend toward a denser state [44].

## 4. Conclusions

Drawing on the Yongjin Expressway reconstruction and expansion project, this study systematically investigated the durability comparison of lime- and cement-improved argillaceous siltstone fill. Through unconfined compressive strength, California bearing ratio, and five wet–dry cycle tests, the difference laws in strength development, deformation characteristics, water stability performance, and durability evolution between the two improvement schemes were quantitatively revealed. The main conclusions are as follows:

(1) Significant differences exist in strength development and improvement efficiency. Under identical dosage (3–7%) and curing conditions, the UCS and CBR of cement-improved soil are significantly higher than those of lime-improved soil. At the same dosage, cement strength is approximately 1.5–1.7 times that of lime, and the absolute strength gap further widens with increasing dosage. The strength enhancement efficiency of cement hydration (approximately 126–137 kPa/%) is far higher than that of pozzolanic reactions (92 kPa/%), but the CBR incremental efficiency of lime improvement shows better sustainability in the high-dosage range.
(2) Both satisfy volume stability engineering control requirements, but cement is superior. Both stabilizers can effectively inhibit the immersion swelling of argillaceous siltstone, but the swelling rate of lime-improved soil is approximately 1.3–1.5 times that of cement-improved soil at the same dosage. After increasing dosage to 7%, the swelling rates of cement soil and lime soil decrease to 0.40% and 0.60%, respectively, both far below the subgrade fill swelling rate control threshold, meeting the volume stability requirements for high-grade highway subgrades.
(3) Wet–dry cycle durability shows dosage-sensitive differentiation. After five wet–dry cycles, the UCS retention rate of 7% cement-improved soil is 78.3%, and that of lime-improved soil is 69.0%; residual strengths are 507.0 kPa and 303.6 kPa, respectively, both satisfying general subgrade engineering strength requirements. However, the UCS of 3% lime-improved soil drops to 47.5 kPa after cycling, falling below the engineering usability threshold, with insufficient long-term stability.

This study is primarily based on 7-day standard curing age and five wet–dry cycle test conditions, and cannot fully reveal the strength evolution laws and micro-structural deterioration mechanisms of the two stabilizers under long-term service environments (such as 180-day curing age, more cycle numbers). Future research may combine scanning electron microscopy (SEM), X-ray diffraction (XRD), and nuclear magnetic resonance (NMR) micro-testing methods to deeply track the sequential evolution characteristics of pozzolanic and hydration reactions, and establish strength prediction models considering the coupled effects of cycle numbers and curing age, providing more refined theoretical support for the full life-cycle design of argillaceous siltstone subgrades.

## Figures and Tables

**Table 1:** Material parameter of argillaceous siltstone.

Optimum Moisture Content	Maximum Dry Density	Liquid Limit	Plastic Limit	Plasticity Index
18.09%	1.70 g/cm^3^	45%	25%	20

**Table 2:** Test scheme design.

Group	Stabilizer Type	Dosage (%)	Optimum Moisture Content (%)	Maximum Dry Density (g/cm^3^)
Plain soil	None	0	18.09	1.70
A1	Lime	3	19.74	1.74
A2		5	20.86	1.77
A3		7	22.03	1.79
B1	Cement	3	22.23	1.79
B2		5	23.41	1.80
B3		7	24.92	1.86

## Data Availability

The original contributions presented in the study are included in the article, further inquiries can be directed to the corresponding author.
